# Is having a 20-minute neighbourhood associated with eating out behaviours and takeaway home delivery? A cross-sectional analysis of ProjectPLAN

**DOI:** 10.1186/s12889-022-12587-1

**Published:** 2022-01-28

**Authors:** Laura H. Oostenbach, Karen E. Lamb, Lukar E. Thornton

**Affiliations:** 1 Institute for Physical Activity and Nutrition (IPAN), School of Exercise and Nutrition Sciences, Deakin University, Geelong, Victoria Australia; 2 Melbourne School of Population and Global Health, University of Melbourne, Parkville, Australia; 3 Department of Marketing, Faculty of Business and Economics, University of Antwerp, Antwerp, Belgium

**Keywords:** Food practices, Out-of-home foods, Neighbourhood, 20-min neighbourhood, Australia

## Abstract

**Background:**

Through improved service provision and accessibility, 20-min neighbourhoods (20MNs) aim to enable people to meet most of their daily (non-work) needs within 20 min from home. Associations between 20MNs and food practices remain unknown. This study examines links with the frequency and location of eating out behaviours as well as the frequency of home food delivery.

**Methods:**

This cross-sectional study used data from 769 adults from the Places and Locations for Activity and Nutrition study (ProjectPLAN) conducted in Melbourne and Adelaide, Australia, between 2018 and 2019. Outcomes were 1) visit frequency to i) cafés, ii) restaurants, bars or bistros, iii) major chain fast food outlets and iv) takeaway outlets to purchase food; 2) total number of different types of out-of-home food outlets visited; 3) use frequency of home food delivery services; 4) distance from home to the most frequented out-of-home food outlets. Exposure was whether participants had a 20MN (areas with high service/amenity provision) or a non-20MN (areas with low service/amenity provision). Ordinal regression models were fitted for the frequency outcomes. Poisson regression models were fitted for the number of different outlet types. Linear and spatial regression models were fitted for the distance outcomes.

**Results:**

Results suggested no differences in frequency of visitations to out-of-home food outlets and use of food delivery services between those with a 20MN and those with a non-20MN. Yet, those with a 20MN were more likely to use a greater number of different types of outlets on a weekly basis. Where a regular eating out location was reported, it was nearer to home for those with a 20MN.

**Conclusions:**

This study provides evidence supportive of 20MNs potentially facilitating more localised food practices, however, 20MNs may also encourage greater cumulative frequency of meals out across a variety of out-of-home food providers.

**Supplementary Information:**

The online version contains supplementary material available at 10.1186/s12889-022-12587-1.

## Background

The recent rapid increase in the size of urban populations worldwide has necessitated a focus on creating liveable cities [1, 2]. Various cities across the world have developed plans in response to population growth (e.g. [3–5],). One concept increasingly included in urban plans is that of compact city designs branded as 20-min neighbourhoods (20MNs) [3, 6]. This concept which originated in Portland (United States (US)) holds local accessibility of services and amenities as key to neighbourhood liveability [3]. Interest in 20MNs has grown in recent years, with cities such as Melbourne (Australia) [6, 7], Paris (France) [5], Edinburgh, (United Kingdom) [8], and Tempe (US) [4] incorporating similar concepts in their urban plans.

In Melbourne, the creation of 20MNs was put forward as a key part of a strategy to accommodate urban growth and ensure liveable neighbourhoods [6, 7]. Through improved service provision and accessibility, 20MNs aim to enable people to meet most of their daily (non-work-related) needs within 20 min from home. Whilst untested environmental, social, economic, and health benefits have been projected [7], it is also unknown whether benefits of having a 20MN expand to behaviours such as food practices.

Out-of-home foods have become a main component of diets in many high-income countries [9, 10], including Australia [11]. For example, recent food expenditure and food habit trends show that eating out-of-home foods is on the rise in Australia [11]. Home-prepared foods have been partially replaced by foods prepared away from home such as fast food and takeaway meals [12]. These patterns are reflected in household expenditure trends, with Australian households spending 34% of their food budget on out-of-home foods in 2015–16 compared to 27.5% in 2003–04 [11]. Out-of-home foods tend to be more energy-dense and nutrient-poor than foods prepared at home, potentially contributing to poorer diet quality [13]. Overall, Australia’s nutritional health status is poor, with most of the population generally failing to meet dietary guidelines [14]. Other high-income countries follow similar diet quality trends [15, 16]. Understanding drivers of out-of-home food consumption is therefore essential to help improve population diet, particularly when poorer diets are key contributors to chronic conditions such as obesity, type II diabetes and cardiovascular disease [17, 18].

When considering the evidence on the association between neighbourhood factors (such as food accessibility) and food practices, recent reviews have highlighted inconsistencies in findings [19–22]. Studies have mostly focused on the purchasing and consumption of foods from a limited category of food outlet types (e.g., fast food outlets) and few have examined food purchasing locations [23–28]. Ignoring where people buy foods means existing evidence does not reflect how people engage with their neighbourhood. Failing to capture actual interactions (i.e., what, where and how often food is purchased) limits our ability to draw links between neighbourhood and food practices, and, in turn, limits our ability to fully assess implications for health. Besides these practices which require engagement with the neighbourhood, there are additional food behaviours that people can do from home such as ordering food delivery, particularly with the surge in home food delivery options [29–33] that have increased the availability and accessibility of food options including those further away from home. A deeper understanding of how home food delivery services are used in relation to people’s residential neighbourhood is necessary to avoid misrepresenting associations between neighbourhoods and out-of-home food purchasing frequency. Further highlighting the importance of investigating the use of home food delivery options are concerns for public health nutrition [32], with studies indicating (online) food delivery options are mostly unhealthy [29, 34].

This study examines associations between 20MNs and the frequency and location of eating out behaviours as well as the frequency of home food delivery in Melbourne where the 20MN concept has been adopted and in Adelaide, a smaller, less densely populated Australian city, where similar compact and walkable neighbourhoods are proposed in urban plans [35]. We hypothesise that those with a 20MN less frequently visit fast food and takeaway outlets due to the availability of alternative (fresh food for preparation at home) options [36] but more often visit cafés and restaurants which allow for opportunities for social interactions [37, 38]. We also hypothesise that those with a non-20MN more frequently order home delivery for takeaway foods, since those with a non-20MN may have reduced access to food outlets within walkable distances, limiting opportunities to easily visit food outlets or pick up takeaway. Complementary to this, we expect those with a 20MN and frequently visiting out-of-home food outlets to do so closer to home compared to those with a non-20MN.

## Methods

### Recruitment

This cross-sectional study used data from the Places and Locations for Activity and Nutrition study (ProjectPLAN), conducted between October 2018 and May 2019 among adults living in Melbourne and Adelaide, Australia. ProjectPLAN was designed to investigate the benefits of having a 20MN on physical activity and food behaviours. Ethics approval was obtained from the Deakin University Human Research Ethics Committee (HEAG-H 168_2017).

### Participant recruitment

Participants were randomly sampled from within three strata: city (Melbourne or Adelaide), neighbourhood status (20MN or non-20MN, see definition below) and neighbourhood socioeconomic status (SES) (low or high), only considering address points within residential Mesh Blocks (which are the smallest geographical areas defined by the Australian Bureau of Statistics (ABS)) to reduce mails out to non-residential addresses. In each city, address points with a 20MN or non-20MN were considered for sampling if they had either low or high neighbourhood SES. Neighbourhood SES was based on the 2016 ABS Socio-Economic Indexes for Areas (SEIFA) Index of Relative Socio-economic Advantage and Disadvantage (IRSAD) [39]. The IRSAD summarises information about the economic and social conditions of people and households within an area, including both relative advantage and disadvantage measures, based on information from the Australian census, including income and occupation [40]. Low SES areas were based on the Statistical Areas level 1 (SA1) SEIFA IRSAD decile 1, 2 or 3 that had to be within larger statistical areas (SA2s) of decile 1, 2 or 3. SA1s within SA2 boundaries were extracted to represent small areas of low SES within a larger community that also had low SES. High SES was classified as SA1s with a SEIFA IRSAD decile of 8, 9 or 10 within an SA2 of decile 8, 9 or 10 [40].

A random selection of households within the chosen areas were invited by mail with an invitation letter including a URL and unique password to access the survey. When accessing the survey, participants were provided with a Plain Language Statement and consent form. Separate surveys were conducted for the food and physical activity behaviours. An adult (aged > 18 years) responsible for the majority of food shopping for the household was asked to complete the food behaviours survey.

### Measures

#### Outcomes

The study examined 1) the usual frequency of visits to each of i) cafés, ii) restaurants, bars or bistros, iii) major chain fast food outlets such as McDonald’s, KFC, Subway, Domino’s (representing chain brand and mostly quick serve options) and iv) other (mostly independent) takeaway outlets such as sushi, Thai, Mexican, fish and chips to purchase food (responses coded: never or less than once every 2 weeks, once every 2 weeks, at least once per week); 2) the total number of different types of food outlets (cafés, restaurants/bars/bistros, major chain fast food outlets, other takeaway outlets) usually frequented at least once per week (range: 0–4); 3) the usual frequency of takeaway home deliveries (coded: never or less than once every 2 weeks, once every 2 weeks, at least once per week); and 4) the distance from home to the most frequently used i) café, ii) restaurant, bar or bistro, iii) major chain fast food outlet, and iv) takeaway outlet. Distance outcomes were only captured for participants who frequented an outlet at least once every 2 weeks and who provided details of the most frequently visited outlet. Participants were asked to provide the name of the outlet as well as the suburb and street name or nearest intersection or landmark for this food outlet. Full addresses were obtained using Google Maps and the X-Y coordinates were extracted. Participant home addresses were mapped and the shortest road network distance between the home address and the outlet was calculated for each participant, using the osrmtime user-written command in Stata [41]. This command calculates the distance between two points based on latitude and longitude data, using the Open Source Routing Machine (OSRM) and OpenStreetMap [41]. Distances greater than 40 km were deemed to be outliers and excluded from the analysis for each outcome.

#### Exposure

The exposure was whether the participant had a 20MN or a non-20MN. Twenty-minute neighbourhoods were areas that had access to a select list of services and amenities grouped within five domains: 1) healthy food (a large supermarket within a 1.5 km pedestrian network distance from home OR a smaller supermarket AND greengrocer (i.e. fruit and vegetable outlet/market) within a 1.5 km pedestrian network distance from home); 2) recreational resources (a gym within a 1.5 km pedestrian network distance from home); 3) community resources (a primary school AND general practitioner (GP) AND pharmacy AND library AND post office AND café within a 1.5 km pedestrian network distance from home); 4) public open space (access to public open space within 400 m pedestrian network distance from home AND ≥ 8 ha of public open space within 1 km from home); and 5) public transport (Melbourne within 5 km of GPO (General Post Office): a bus stop within a 400 m pedestrian network distance from home OR a tram stop within a 600 m pedestrian network distance from home OR a train station within an 800 m pedestrian network distance from home; Melbourne further than 5 km from the GPO: a train station within an 800 m pedestrian network distance from home AND either a bus stop within a 400 m pedestrian network distance from home OR a tram stop within a 600 m pedestrian network distance from home; Adelaide: a bus or O-Bahn stop within a 400 m pedestrian network distance from home OR a tram stop within a 600 m pedestrian network distance from home OR a train station within an 800 m pedestrian network distance from home). Non-20MNs had fewer than five of the individual services and amenities in Melbourne and fewer than four of the individual services and amenities in Adelaide due to the different public transport measures used across the two cities. Full details of the operationalisation of the 20MN have been published elsewhere [42].

#### Other covariates

Age (years), gender (male, female), presence of children in the household (no children, presence of at least one child < 4 years, only children aged 5 to 17 years), highest qualification (less than university, university), ability to manage on household income (very difficult/difficult, just getting by, comfortable/very comfortable), neighbourhood SES (low, high), and neighbourhood self-selection (not within 20MN/in 20MN but not important, in 20MN and important) considered as potential confounders.

Neighbourhood self-selection relates to people choosing to live in a neighbourhood with facilities and resources that accommodate their preferred lifestyles [43]. Adjusting for neighbourhood self-selection can therefore help distinguish the impact of neighbourhood features on behaviours from the choice to live near features facilitating those preferred behaviours [43]. Choosing to live in a neighbourhood that met their everyday (non-work) needs within a 20-min walk was used as a measure of neighbourhood self-selection in this study.

### Statistical analysis

Ordinal regression models were fitted for each frequency outcome. Likelihood ratio tests were used to compare constrained (i.e., assuming proportional odds) and unconstrained models to verify the proportional odds assumption. Minimally adjusted models included only the stratification variable neighbourhood SES. Adjusted models included all potential confounders. Models with and without adjustment for neighbourhood self-selection were fitted to examine the impact of self-selection adjustment on findings. Poisson regression models were fitted for the number of different outlet types frequented at least once per week.

A complete case analysis was conducted assuming data were Missing Completely At Random (MCAR). Sample characteristics for the complete case sample appeared to be representative of the original sample (see Additional file 1). Sample sizes for each outcome are shown in Additional file 2.

Additionally, linear regression models were fitted to examine differences in mean distance travelled to each food outlet type by 20MN, adjusting for neighbourhood SES. Adjusted models included age, gender and neighbourhood self-selection. Distances were log-transformed to deal with the skewed distributions and coefficients and confidence intervals were exponentiated to obtain the geometric mean ratio (GMR). Unlike the arithmetic mean which is estimated by summing the n observations and dividing by n, geometric means are calculated as the n^th^ root of the product of n numbers (e.g., the geometric mean of the two numbers 9 and 4 is the square root of 36 [i.e., √(9 × 4)]). Geometric means are a robust way of assessing the central tendency of a data set when dealing with skewed data. Fitting a linear regression to an untransformed outcome provides estimates of the arithmetic mean and differences in arithmetic means. However, when an outcome is log-transformed to deal with the skewed nature of the distribution, the exponentiated coefficients are estimated geometric means and GMRs. As with odds or risk ratios, the null value for a GMR is 1; values above 1 indicate a percentage increase as the exposure variable increases (e.g., a GMR of 1.12 would indicate a 12% increase with each unit increase in the continuous exposure variable), while values less than 1 indicate a percentage decrease (e.g., a GMR of 0.75 would indicate a 25% decrease with each unit increase in the continuous exposure variable). The final sample sizes for each outcome are shown in Additional file 3.

Distance travelled was assumed to be potentially more correlated for participants residing closer to one another. Therefore, an inverse distance matrix was created using participants’ home address in each city and the Moran’s I test [44] was used to test for evidence of global residual spatial autocorrelation in the adjusted models. Moran’s I is the most commonly used measure of spatial autocorrelation, providing a measure of how related observations are (i.e., model residuals in this context) based on the location at which they are measured. It is a measure of correlation which ranges from values of − 1 which indicates negative spatial autocorrelation or complete spatial dispersion to 1 indicating high positive spatial autocorrelation. A value of 0 indicates complete spatial randomness (perfect dispersion). The Moran’s I test statistic (i.e., the estimated spatial autocorrelation) is assumed to follow a Χ^2^ distribution with 1 degree of freedom in the Moran’s I test. This was conducted with Stata command estat moran. Where there was evidence of residual spatial autocorrelation, spatial autoregressive models were fitted with Stata command spregress to include spatially lagged errors using the inverse distance matrix.

All analyses were conducted separately for each city using Stata version 16.0.

## Results

### Descriptive characteristics

In total, 769 participants completed the food survey (Melbourne = 358; Adelaide = 411, see Additional file 2). Of these, 306 (85.5%) from Melbourne and 374 (91.0%) from Adelaide were included in the complete case analysis. Sample characteristics are detailed in Table 1.

**Table 1:** Descriptive characteristics of the participants by city and 20MN status (*N* = 680)

	Melbourne			Adelaide		
	Overall	20-min neighbourhood	Non-20-min neighbourhood	Overall	20-min neighbourhood	Non-20-min neighbourhood
	***N*** = 306	***N*** = 136	***N*** = 170	***N*** = 374	***N*** = 198	***N*** = 176
**Frequency of visits to cafes**						
Less than once per fortnight	118 (38.6%)	41 (30.1%)	77 (45.3%)	179 (47.9%)	84 (42.4%)	95 (54.0%)
Once per fortnight	40 (13.1%)	16 (11.8%)	24 (14.1%)	49 (13.1%)	27 (13.6%)	22 (12.5%)
At least once per week	148 (48.4%)	79 (58.1%)	69 (40.6%)	146 (39.0%)	87 (43.9%)	59 (33.5%)
**Frequency of visits to restaurants/bistros/bars**						
Less than once per fortnight	167 (54.6%)	61 (44.9%)	106 (62.4%)	217 (58.0%)	106 (53.5%)	111 (63.1%)
Once per fortnight	67 (21.9%)	33 (24.3%)	34 (20.0%)	72 (19.3%)	39 (19.7%)	33 (18.8%)
At least once per week	72 (23.5%)	42 (30.9%)	30 (17.6%)	85 (22.7%)	53 (26.8%)	32 (18.2%)
**Frequency of visits to major chain fast food outlets**						
Less than once per fortnight	224 (73.2%)	101 (74.3%)	123 (72.4%)	292 (78.1%)	155 (78.3%)	137 (77.8%)
Once per fortnight	41 (13.4%)	15 (11.0%)	26 (15.3%)	37 (9.9%)	19 (9.6%)	18 (10.2%)
At least once per week	41 (13.4%)	20 (14.7%)	21 (12.4%)	45 (12.0%)	24 (12.1%)	21 (11.9%)
**Frequency of visits to takeaway outlets**^**a**^						
Less than once per fortnight	192 (62.7%)	83 (61.0%)	109 (64.1%)	249 (66.8%)	131 (66.5%)	118 (67.0%)
Once per fortnight	54 (17.6%)	19 (14.0%)	35 (20.6%)	68 (18.2%)	32 (16.2%)	36 (20.5%)
At least once per week	60 (19.6%)	34 (25.0%)	26 (15.3%)	56 (15.0%)	34 (17.3%)	22 (12.5%)
**Number of types of out-of-home outlets visited at least once per week**						
0	112 (36.6%)	39 (28.7%)	73 (42.9%)	170 (45.6%)	82 (41.6%)	88 (50.0%)
1	101 (33.0%)	45 (33.1%)	56 (32.9%)	106 (28.4%)	54 (27.4%)	52 (29.5%)
2	64 (20.9%)	31 (22.8%)	33 (19.4%)	68 (18.2%)	42 (21.3%)	26 (14.8%)
3	24 (7.8%)	16 (11.8%)	8 (4.7%)	26 (7.0%)	16 (8.1%)	10 (5.7%)
4	5 (1.6%)	5 (3.7%)	0 (0.0%)	3 (0.8%)	3 (1.5%)	0 (0.0%)
**Frequency of takeaway deliveries**^**a**^						
Less than once per fortnight	265 (86.6%)	112 (82.4%)	153 (90.0%)	349 (93.6%)	183 (92.4%)	166 (94.9%)
Once per fortnight	22 (7.2%)	11 (8.1%)	11 (6.5%)	14 (3.8%)	8 (4.0%)	6 (3.4%)
At least once per week	19 (6.2%)	13 (9.6%)	6 (3.5%)	10 (2.7%)	7 (3.5%)	3 (1.7%)
**Neighbourhood SES**						
Low SES	136 (44.4%)	50 (36.8%)	86 (50.6%)	158 (42.2%)	64 (32.3%)	94 (53.4%)
High SES	170 (55.6%)	86 (63.2%)	84 (49.4%)	216 (57.8%)	134 (67.7%)	82 (46.6%)
**Age (years), mean (SD)**	51.9 (15.8)	49.6 (16.3)	53.7 (15.2)	56.5 (15.7)	56.3 (16.5)	56.6 (14.8)
**Gender**						
Male	122 (39.9%)	54 (39.7%)	68 (40.0%)	145 (38.8%)	74 (37.4%)	71 (40.3%)
Female	184 (60.1%)	82 (60.3%)	102 (60.0%)	229 (61.2%)	124 (62.6%)	105 (59.7%)
**Highest qualification**						
Less than university	120 (39.2%)	34 (25.0%)	86 (50.6%)	209 (55.9%)	95 (48.0%)	114 (64.8%)
University	186 (60.8%)	102 (75.0%)	84 (49.4%)	165 (44.1%)	103 (52.0%)	62 (35.2%)
**Children in household**						
No children	206 (67.3%)	101 (74.3%)	105 (61.8%)	293 (78.3%)	153 (77.3%)	140 (79.5%)
At least one child < 4 yrs	54 (17.6%)	22 (16.2%)	32 (18.8%)	40 (10.7%)	25 (12.6%)	15 (8.5%)
Only child (ren) 5–17 yrs	46 (15.0%)	13 (9.6%)	33 (19.4%)	41 (11.0%)	20 (10.1%)	21 (11.9%)
**Ability to manage on income**						
Very difficult/difficult	30 (9.8%)	15 (11.0%)	15 (8.8%)	43 (11.5%)	21 (10.6%)	22 (12.5%)
Just getting by	71 (23.2%)	27 (19.9%)	44 (25.9%)	85 (22.7%)	44 (22.2%)	41 (23.3%)
Comfortable/Very comfortable	205 (67.0%)	94 (69.1%)	111 (65.3%)	246 (65.8%)	133 (67.2%)	113 (64.2%)
**Everyday needs within 20 min and reason for moving/living here**						
No, not within 20 min/Yes but not important	149 (48.7%)	28 (20.6%)	121 (71.2%)	193 (51.6%)	64 (32.3%)	129 (73.3%)
Yes and important	157 (51.3%)	108 (79.4%)	49 (28.8%)	181 (48.4%)	134 (67.7%)	47 (26.7%)

^a^Note: 1 person in Adelaide had missing takeaway outlet visits and delivery frequency information

### Frequency of visits to out-of-home food outlets, number of types of weekly frequented outlets, and frequency of takeaway home deliveries

Compared with participants in a non-20MN, a higher proportion of participants with a 20MN visited cafes (Melbourne 58% vs 41%, Adelaide 44% vs 34%), restaurants/bistros/bars (Melbourne 31% vs 18%, Adelaide 27% vs 18%) and takeaway outlets (Melbourne 25% vs 15%, Adelaide 17% vs 13%) at least once per week. There was little difference in the distribution of frequency of visits to major chain fast food outlets by 20MNs vs non-20MNs for either city. A smaller proportion of participants with a 20MN did not visit any food outlet at least once per week (Melbourne 29%, Adelaide 42%) than with a non-20MN (Melbourne 43%, Adelaide 50%). Only a small proportion of participants ordered takeaway delivery at least once per week, with the majority ordering less than once per fortnight (Table 1).

Although the results from the minimally adjusted models suggested increased frequency of visitations to cafés and restaurants for participants with a 20MN compared to a non-20MN in Melbourne (Table 2), the magnitude of effect attenuated after adjustment for confounders (Cafes: odds ratio (OR) = 1.51, 95% confidence interval (CI) 0.88–2.59; Restaurants: OR = 1.19, 95% CI 0.70–2.03). There did not appear to be differences in frequency of visitation to major chain fast food outlets or takeaway outlets between 20MNs and non-20MNs in Melbourne (Table 2). In Adelaide, there was no evidence of a difference in frequency of visits between 20MNs and non-20MNs for any outlet type.

**Table 2:** Models of the frequency outcomes and the number of food outlet types visited

	Melbourne ***N*** = 306									Adelaide ***N*** = 374^*^								
	Minimally adjusted^**1**^			Adjusted without self-selection^**2**^			Adjusted with self-selection^**3**^			Minimally adjusted^**1**^			Adjusted without self-selection^**2**^			Adjusted with self-selection^**3**^		
	**OR**	**95% CI**	***p*-value**	**OR**	**95% CI**	***p*-value**	**OR**	**95% CI**	***p*-value**	**OR**	**95% CI**	***p*-value**	**OR**	**95% CI**	***p*-value**	**OR**	**95% CI**	***p*-value**
**Cafes**																		
20MN	1.82	(1.16, 2.83)	0.008	1.47	(0.91, 2.36)	0.116	1.51	(0.88, 2.59)	0.138	1.30	(0.87, 1.95)	0.200	1.31	(0.87, 1.97)	0.195	1.32	(0.85, 2.05)	0.220
**Restaurants**																		
20MN	1.97	(1.27, 3.06)	0.003	1.53	(0.95, 2.45)	0.077	1.19	(0.70, 2.03)	0.520	1.34	(0.89, 2.02)	0.161	1.43	(0.93, 2.19)	0.100	1.42	(0.89, 2.29)	0.143
**Major chain fast food**																		
20MN	1.02	(0.61, 1.70)	0.951	1.03	(0.58, 1.84)	0.914	0.86	(0.45, 1.65)	0.655	1.27	(0.76, 2.13)	0.355	1.32	(0.75, 2.32)	0.332	1.40	(0.77, 2.55)	0.270
**Takeaway**																		
20MN	1.28	(0.81, 2.02)	0.297	1.19	(0.71, 1.99)	0.508	1.30	(0.72, 2.35)	0.376	1.13	(0.73, 1.74)	0.580	1.11	(0.71, 1.74)	0.639	1.10	(0.68, 1.79)	0.687
**Takeaway delivery**																		
20MN	1.87	(0.95, 3.67)	0.069	1.43	(0.68, 3.03)	0.343	0.98	(0.42, 2.26)	0.958	1.84	(0.76, 4.41)	0.175	1.65	(0.61, 4.45)	0.321	2.04	(0.72, 5.82)	0.182
	**IRR**	**95% CI**	***p*-value**	**IRR**	**95% CI**	***p*-value**	**IRR**	**95% CI**	***p*-value**	**IRR**	**95% CI**	***p*-value**	**IRR**	**95% CI**	***p*-value**	**IRR**	**95% CI**	***p*-value**
**Number of outlet types visited at least once per week**																		
20MN	1.47	(1.18, 1.84)	0.001	1.35	(1.06, 1.70)	0.013	1.29	(0.99, 1.69)	0.058	1.27	(1.01, 1.59)	0.038	1.28	(1.02, 1.61)	0.034	1.25	(0.98, 1.61)	0.076

Ordinal and Poisson regression models of the frequency of visits to each food outlet, frequency of food delivery use, and the number of types of outlets visited at least once per week by 20MN status and city

^1^Adjusted for neighbourhood socioeconomic status

^2^Adjusted for neighbourhood socioeconomic status, age, gender, qualifications, children in the household, ability to manage on income

^3^Adjusted for neighbourhood socioeconomic status, age, gender, qualifications, children in the household, ability to manage on income, neighbourhood self-selection

^*^N = 373 for takeaway outlets, takeaway delivery and number of outlet types in Adelaide

When examining the total number of different outlet types visited, participants with a 20MN were more likely to visit a greater number of types at least once per week than those with a non-20MN (Table 2), although the magnitude of the effect attenuated after adjustment for confounders (Melbourne: incidence rate ratio (IRR) = 1.29, 95% CI 0.99–1.69; Adelaide: IRR = 1.25, 95% CI 0.98–1.61). No differences in the frequency of takeaway home delivery were observed between 20MNs and non-20MNs across both cities.

### Distance travelled to most visited café, restaurant/bistro/bar, major chain fast food outlet and takeaway outlet

**Fig. 1:**
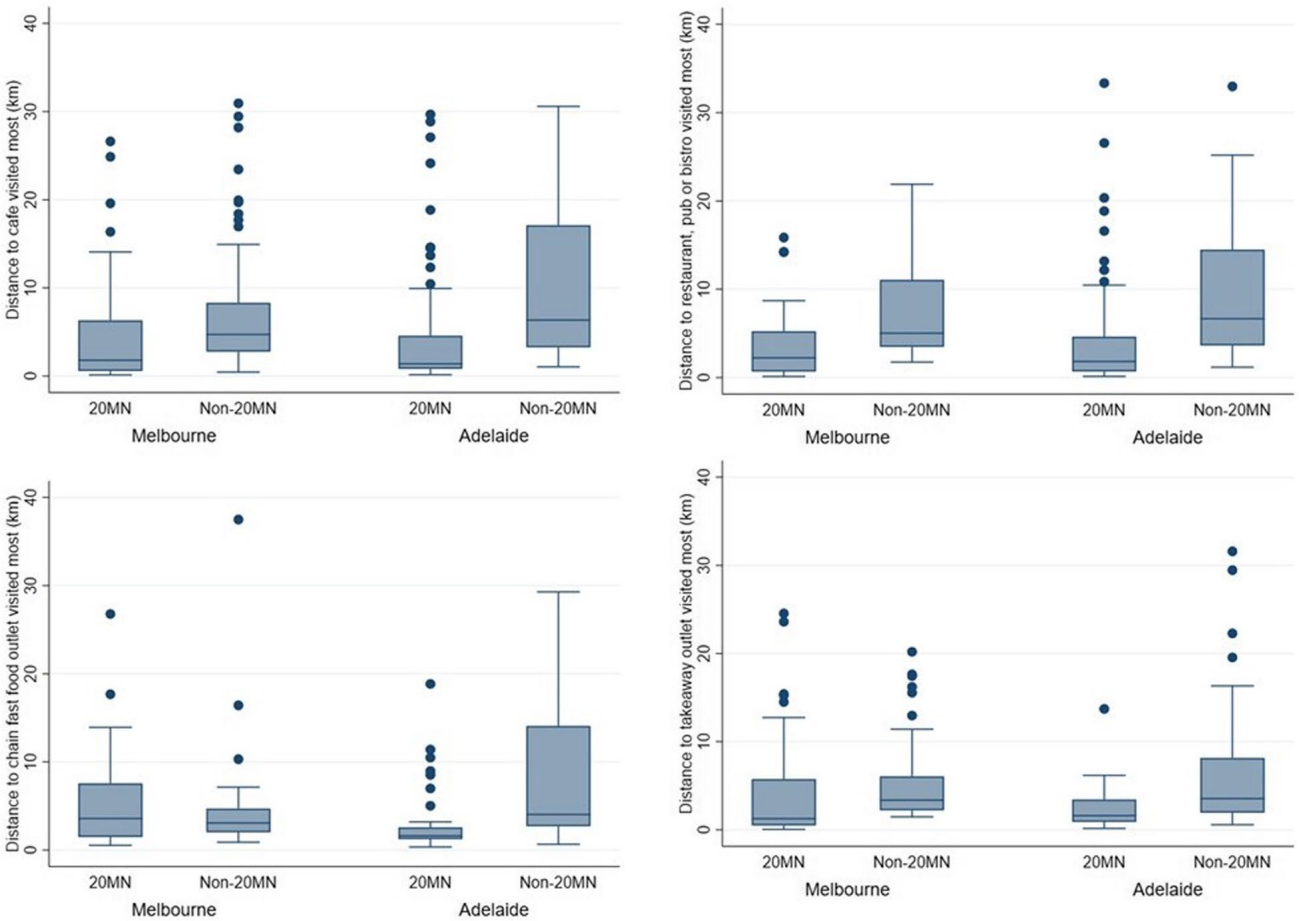
Boxplots of the distance to the most frequented food outlet by city and neighbourhood status. Legend: Sample sizes differ for each outlet as not all participants regularly visit each of these outlets. Café: Melbourne: *N* = 157; Adelaide: *N* = 175. Restaurant/bistro/bar: Melbourne: *N* = 98; Adelaide: *N* = 134. Major chain fast food outlet: Melbourne: *N* = 68; Adelaide: *N* = 74. Takeaway outlet: Melbourne: *N* = 94; Adelaide: *N* = 103

The median distance travelled to the most frequented café was much lower for those with a 20MN (Melbourne: 1.8 km, Adelaide: 1.4 km) than those with a non-20MN (Melbourne: 4.7 km, Adelaide: 6.3 km) (Fig. 1 and Additional file 4). This was also the case for restaurants, bars or bistros and takeaway outlets. The median distance to the major chain fast food outlet most frequented was higher for participants with a 20MN (3.6 km) than a non-20MN (3.1 km) in Melbourne. However, this was not the case in Adelaide (20MN: 1.6 km, non-20MN: 4.0 km).

Results from the linear regression models suggested that those with a 20MN in Melbourne travelled between 49 and 59% shorter distances to visit their most frequented café, restaurant or takeaway than those with a non-20MN, after accounting for age, gender and self-selection (Table 3). The evidence did not suggest a difference in distance to major chain fast food outlets between 20MNs and non-20MNs (Adjusted GMR = 1.11, 95% CI 0.69–1.77).

**Table 3:** Models of the difference in log-distances to each food outlet

	Melbourne									Adelaide								
	Minimally adjusted^**a**^			Adjusted without self-selection^**b**^			Adjusted with self-selection^**c**^			Minimally adjusted^**d**^			Adjusted without self-selection^**b**^			Adjusted with self-selection^**c**^		
	GMR	95% CI	***p***-value	GMR	95% CI	***p***-value	GMR	95% CI	***p***-value	GMR	95% CI	***p***-value	GMR	95% CI	***p***-value	GMR	95% CI	***p***-value
**Cafes**^**d**^																		
20MN	0.43	(0.30, 0.61)	< 0.001	0.43	(0.29, 0.62)	< 0.001	0.49	(0.31, 0.78)	0.003	0.31	(0.22, 0.43)	< 0.001	0.32	(0.23, 0.44)	< 0.001	0.31	(0.21, 0.44)	< 0.001
**Restaurants**^**e**^																		
20MN	0.44	(0.29, 0.65)	< 0.001	0.42	(0.28, 0.63)	< 0.001	0.41	(0.25, 0.66)	< 0.001	0.31	(0.22, 0.43)	< 0.001	0.31	(0.20, 0.46)	< 0.001	0.35	(0.22, 0.55)	< 0.001
**Major chain fast food**^**f**^																		
20MN	1.06	(0.70, 1.62)	0.769	1.00	(0.64, 1.56)	0.990	1.11	(0.69, 1.77)	0.664	0.33	(0.21, 0.53)	< 0.001	0.33	(0.20, 0.52)	< 0.001	0.33	(0.20, 0.54)	< 0.001
**Takeaway**^**g**^																		
20MN	0.42	(0.26, 0.68)	0.001	0.43	(0.26, 0.71)	0.001	0.51	(0.28, 0.93)	0.028	0.38	(0.26, 0.55)	< 0.001	0.37	(0.26, 0.54)	< 0.001	0.40	(0.26, 0.60)	< 0.001

*GMR* Geometric Mean Ratio

^a^Adjusted for neighbourhood socioeconomic status

^b^Adjusted for neighbourhood socioeconomic status, age and gender

^c^Adjusted for neighbourhood socioeconomic status, age, gender, neighbourhood self-selection

^d^Melbourne: *N* = 157; Adelaide: *N* = 175

^e^Melbourne: *N* = 98; Adelaide: *N* = 134

^f^Melbourne: *N* = 68; Adelaide: *N* = 74

^g^Melbourne: *N* = 94; Adelaide: *N* = 103

In Adelaide, there was strong evidence that those with a 20MN travelled shorter distances to visit their most frequented outlet for all food outlet types (Table 3). Results showed participants with a 20MN travelled 69% (95% CI 56–79%) shorter distances to cafes, 65% (95% CI 45–78%) shorter distances to restaurants/bars/bistros, 67% (95% CI 46–80%) shorter distances to major chain fast food outlets and 60% (95% CI 40–74%) shorter distances to takeaway outlets.

The Moran’s I tests showed strong evidence of residual spatial autocorrelation in the models of log-distance to the most frequently visited café and takeaway in Melbourne (Table 4). The findings from spatial regression models were similar to the results from linear regression models for these outcomes (Cafes: GMR = 0.42, 95% CI 0.27–0.65, *p* < 0.001; Restaurants: GMR = 0.48, 95% CI 0.29–0.80, *p* = 0.005).

**Table 4:** Moran’s I test of residual spatial autocorrelation for linear regression models^a^ of log-distance outcomes

	Melbourne		Adelaide	
	Χ^**2**^	***p***-value	Χ^**2**^	***p***-value
Cafes	12.96	< 0.001	2.56	0.110
Restaurants/bars/bistros	0.20	0.654	0.05	0.826
Major chain fast food	1.39	0.238	0.04	0.837
Takeaway	8.45	0.004	0.18	0.673

^a^Moran’s I test conducted on the model adjusted for neighbourhood socioeconomic status, age, gender and neighbourhood self-selection

## Discussion

The study examined whether 20MNs were associated with the frequency of out-of-home food outlet use, the number of different types used, the location of the most frequently visited outlets, and use of home-delivery services. Results suggested no differences between those with a 20MN and those with a non-20MN in terms of the frequency of visitations to out-of-home food outlets and use of home food delivery services. Yet, results suggest those with a 20MN were more likely to use a greater number of different types of out-of-home food outlets on a weekly basis, and for those that reported a regular out-of-home food outlet they visit, it was nearer to home for those with a 20MN. Thus, 20MNs seem to potentially facilitate more localised food practices, in this case, those related to eating out.

A greater variety of visited out-of-home food outlet types for those with a 20MN is possibly linked to the wider range of choices that are likely available in 20MNs [45]. Another explanation could also relate to some 20MNs having more compact housing to encourage higher population density. In the Australian context this often means smaller housing which may lead to less home cooking if residents feel the kitchens are restrictive and the ability to host guests for meals at home is reduced [46], although home cooking was not assessed in this study.

No differences in the frequency of home delivery of takeaway foods were found. A possible explanation may relate to food delivery services being easily available and accessible regardless of neighbourhood type [29, 32]. Convenience, rather than neighbourhood factors, may be a more important driver of food delivery services usage [31, 47]. While the rapid growth of food delivery providers has driven research into these services [29, 30, 33, 34], further research is needed into the individual determinants of their use and the moderating role of the neighbourhood food environment.

Understanding how different individuals interact with their neighbourhood and where they perform food practices is crucial for building stronger evidence between exposure and use. Except for major chain fast food outlets in Melbourne where no differences were observed (perhaps owing to the high proliferation of chain fast food outlets across all areas [48]), those with a 20MN travelled shorter distances to visit their most frequented outlet, suggesting 20MNs encourage localised food purchasing and interactions with local businesses. Advantages that may stem from the localised use of these outlets are increased opportunities to socialise, build a sense of community, and support local businesses [49].

This study is strengthened by its inclusion of two different Australian cities, allowing for assessment across different contexts, enhancing our understanding of the generalisability of the results. The study is further strengthened by its assessment of multiple out-of-home food outlet types and two behaviours (i.e., visiting food outlets and ordering food delivery). Four different types of out-of-home food outlets as well as home food delivery services were examined, whereas a large majority of studies investigating the relationship between the neighbourhood environment and the use of out-of-home food outlets have mostly been limited in scope to fast food outlets [24, 45, 50–53]. The location of frequently visited outlets was collected with minimum participant burden, using the self-reported address or street intersection and calculating a network distance to this from the participant’s home address. Examining the location of frequented food outlets allows for a more accurate representation of neighbourhood use as it captures where the interaction with the food environment occurs (rather than assuming it is taking place in the most proximal outlets). It should be acknowledged that some sample sizes were small for sub-types of outlets examined in this exploratory analysis as some participants did not regularly visit these outlet types, limiting the statistical power. However, even with these small sample sizes almost all findings were in a consistent direction and effect estimates were of a similar magnitude. Additionally, assessment and adjustment for neighbourhood self-selection helped distinguish the impact of 20MNs on the study behavioural outcomes from the choice to live in a 20MN facilitating those preferred behaviours [43].

However, whilst we were able to examine the most frequented locations, we did not capture location of all outlets visited within a category. Future food environment studies should continue to investigate ways to assess all interactions with the food environment. As frequency of visits to out-of-home food outlets and use of home food delivery services were self-reported, social desirability bias cannot be excluded. For example, frequency of fast food outlet visits may be underreported as fast food consumption may be perceived as socially undesirable. Whilst further targeted mail outs to additional addresses within strata where initial responses were lowest were undertaken (e.g., low SES 20MN addresses), results may not be representative of the wider population.

## Conclusions

This study has provided evidence as to potential links between out-of-home food behaviours and the 20MN design. More specifically, while 20MNs may facilitate more localised out-of-home food practices, they may also encourage greater cumulative frequency of meals out across a variety of food outlet types. Findings are supportive of projected benefits of having a 20MN, i.e., more localised living [7]. The study found no evidence of differences in frequency of visitations to individual out-of-home food outlet types and use of food delivery services between those with a 20MN and those with a non-20MN. More research is needed to assess whether 20MNs promote healthier living.

## Supplementary Information

**Additional file 1.** Descriptive characteristics for the full sample of Project PLAN food survey participants, complete case sample and omitted participants by city*.

**Additional file 2.** Participant flowchart for frequency outcomes.

**Additional file 3.** Participant flowchart for distance outcomes.

**Additional file 4.** Median and inter-quartile range of distances travelled to cafes, restaurants/bars/bistros, major chain fast food outlets and takeaway outlets by city and 20-min neighbourhood status*.

## Data Availability

The datasets used and analysed during the current study are available from the corresponding author on reasonable request.

## Abbreviations

20MNs: 20-min neighbourhoods
US: United States
ProjectPLAN: Project Places and Locations for Activity and Nutrition
SES: Socioeconomic status
ABS: Australian Bureau of Statistics
SEIFA: Socio-Economic Indexes for Areas
IRSAD: Index of Relative Socio-economic Advantage and Disadvantage
SA: Statistical Areas
OSRM: Open Source Routing Machine
GP: General practitioner
GPO: General Post Office
MCAR: Missing completely at random
GMR: Geometric mean ratio
OR: Odds ratio
CI: Confidence interval
IRR: Incidence rate ratio
