# Supplementary material for: Is having a 20-minute neighbourhood associated with eating out behaviours and takeaway home delivery? A cross-sectional analysis of ProjectPLAN
Source: BMC Public Health. 2022 Jan 28;22:191. doi: 10.1186/s12889-022-12587-1 (PMC8796524; doi:10.1186/s12889-022-12587-1)
Supplement: Supplementary file 1 — Additional file 1. Descriptive characteristics for the full sample of Project PLAN food survey participants, complete case sample and omitted participants by city*. [file 12889_2022_12587_MOESM1_ESM.docx]

**Additional file 1**. Descriptive characteristics for the full sample of Project PLAN food survey participants, complete case sample and omitted participants by city*.

|  | **Melbourne Full** | **Melbourne Complete Case** | **Melbourne Omitted** | **Adelaide Full** | **Adelaide Complete Case** | **Adelaide Omitted** |
| --- | --- | --- | --- | --- | --- | --- |
|  | **N = 358** | **N = 306** | **N = 52** | **N = 409^1^** | **N = 374** | **N = 35** |
| **Frequency of visits to cafe** |  |  |  |  |  |  |
| Less than once per fortnight | 140 (39.7%) | 118 (38.6%) | 22 (46.8%) | 194 (47.7%) | 179 (47.9%) | 15 (45.5%) |
| Once per fortnight | 46 (13.0%) | 40 (13.1%) | 6 (12.8%) | 54 (13.3%) | 49 (13.1%) | 5 (15.2%) |
| At least once per week | 167 (47.3%) | 148 (48.4%) | 19 (40.4%) | 159 (39.1%) | 146 (39.0%) | 13 (39.4%) |
| **Frequency of visits to restaurants/bistros/bars** |  |  |  |  |  |  |
| Less than once per fortnight | 198 (56.1%) | 167 (54.6%) | 31 (66.0%) | 236 (58.0%) | 217 (58.0%) | 19 (57.6%) |
| Once per fortnight | 77 (21.8%) | 67 (21.9%) | 10 (21.3%) | 80 (19.7%) | 72 (19.3%) | 8 (24.2%) |
| At least once per week | 78 (22.1%) | 72 (23.5%) | 6 (12.8%) | 91 (22.4%) | 85 (22.7%) | 6 (18.2%) |
| **Frequency of visits to major chain fast food outlets** |  |  |  |  |  |  |
| Less than once per fortnight | 261 (74.1%) | 224 (73.2%) | 37 (80.4%) | 321 (78.9%) | 292 (78.1%) | 29 (87.9%) |
| Once per fortnight | 43 (12.2%) | 41 (13.4%) | 2 (4.3%) | 40 (9.8%) | 37 (9.9%) | 3 (9.1%) |
| At least once per week | 48 (13.6%) | 41 (13.4%) | 7 (15.2%) | 46 (11.3%) | 45 (12.0%) | 1 (3.0%) |
| **Frequency of visits to takeaway outlets** |  |  |  |  |  |  |
| Less than once per fortnight | 218 (61.9%) | 192 (62.7%) | 26 (56.5%) | 276 (68.0%) | 249 (66.8%) | 27 (81.8%) |
| Once per fortnight | 63 (17.9%) | 54 (17.6%) | 9 (19.6%) | 74 (18.2%) | 68 (18.2%) | 6 (18.2%) |
| At least once per week | 71 (20.2%) | 60 (19.6%) | 11 (23.9%) | 56 (13.8%) | 56 (15.0%) | 0 (0.0%) |
| **Frequency of takeaway deliveries** |  |  |  |  |  |  |
| Less than once per fortnight | 306 (86.7%) | 265 (86.6%) | 41 (87.2%) | 382 (94.1%) | 349 (93.6%) | 34 (97.1%) |
| Once per fortnight | 26 (7.4%) | 22 (7.2%) | 4 (8.5%) | 14 (3.4%) | 14 (3.8%) | 1 (2.9%) |
| At least once per week | 21 (5.9%) | 19 (6.2%) | 2 (4.3%) | 10 (2.5%) | 10 (2.7%) | 0 (0.0%) |
| **Number of types of out-of-home outlets visited at least once per week** |  |  |  |  |  |  |
| 0 | 132 (37.5%) | 112 (36.6%) | 20 (43.5%) | 187 (46.1%) | 170 (45.6%) | 17 (51.5%) |
| 1 | 114 (32.4%) | 101 (33.0%) | 13 (28.3%) | 118 (29.1%) | 106 (28.4%) | 12 (36.4%) |
| 2 | 74 (21.0%) | 64 (20.9%) | 10 (21.7%) | 72 (17.7%) | 68 (18.2%) | 4 (12.1%) |
| 3 | 27 (7.7%) | 24 (7.8%) | 3 (6.5%) | 26 (6.4%) | 26 (7.0%) | 0 (0.0%) |
| 4 | 5 (1.4%) | 5 (1.6%) | 0 (0.0%) | 3 (0.7%) | 3 (0.8%) | 0 (0.0%) |
| **Neighbourhood status** |  |  |  |  |  |  |
| 20MN | 158 (44.1%) | 136 (44.4%) | 22 (42.3%) | 218 (53.3%) | 198 (52.9%) | 20 (57.1%) |
| Non-20MN | 200 (55.9%) | 170 (55.6%) | 30 (57.7%) | 191 (46.7%) | 176 (47.1%) | 15 (42.9%) |
| **Neighbourhood SES** |  |  |  |  |  |  |
| Low SES | 162 (45.3%) | 136 (44.4%) | 26 (50.0%) | 173 (42.3%) | 158 (42.2%) | 15 (42.9%) |
| High SES | 196 (54.7%) | 170 (55.6%) | 26 (50.0%) | 236 (57.7%) | 216 (57.8%) | 20 (57.1%) |
| **Age (years), mean (SD)** | 52.5 (15.9) | 51.9 (15.8) | 59.1 (16.2) | 57.1 (15.8) | 56.5 (15.7) | 67.0 (13.9) |
| **Gender** |  |  |  |  |  |  |
| Male | 138 (39.2%) | 122 (39.9%) | 16 (34.8%) | 158 (39.0%) | 145 (38.8%) | 13 (41.9%) |
| Female | 214 (60.8%) | 184 (60.1%) | 30 (65.2%) | 247 (61.0%) | 229 (61.2%) | 18 (58.1%) |
| **Highest qualification** |  |  |  |  |  |  |
| Less than university | 137 (39.1%) | 120 (39.2%) | 17 (38.6%) | 227 (56.5%) | 209 (55.9%) | 18 (64.3%) |
| University | 213 (60.9%) | 186 (60.8%) | 27 (61.4%) | 175 (43.5%) | 165 (44.1%) | 10 (35.7%) |
| **Children in household** |  |  |  |  |  |  |
| No children | 240 (68.0%) | 206 (67.3%) | 34 (72.3%) | 315 (77.6%) | 293 (78.3%) | 22 (68.8%) |
| At least one child < 4 yrs | 62 (17.6%) | 54 (17.6%) | 8 (17.0%) | 49 (12.1%) | 40 (10.7%) | 9 (28.1%) |
| Only child(ren) 5-17 yrs | 51 (14.4%) | 46 (15.0%) | 5 (10.6%) | 42 (10.3%) | 41 (11.0%) | 1 (3.1%) |
| **Ability to manage on income** |  |  |  |  |  |  |
| Very difficult/difficult | 36 (10.5%) | 30 (9.8%) | 6 (15.8%) | 47 (11.8%) | 43 (11.5%) | 4 (16.0%) |
| Just getting by | 84 (24.4%) | 71 (23.2%) | 13 (34.2%) | 95 (23.8%) | 85 (22.7%) | 10 (40.0%) |
| Comfortable/Very comfortable | 224 (65.1%) | 205 (67.0%) | 19 (50.0%) | 257 (64.4%) | 246 (65.8%) | 11 (44.0%) |
| **Everyday needs reason for moving/living here** |  |  |  |  |  |  |
| Not within 20min/Not important | 163 (49.2%) | 149 (48.7%) | 14 (56.0%) | 200 (51.5%) | 193 (51.6%) | 7 (50.0%) |
| Important | 168 (50.8%) | 157 (51.3%) | 11 (44.0%) | 188 (48.5%) | 181 (48.4%) | 7 (50.0%) |

* Note: Missing data proportions are omitted from this table to ease comparison of response proportions across each sample. ^1^Table excludes transgender participants as too few respondents (n=2) for inclusion in analysis.
