# Supplementary figures and images for: Is having a 20-minute neighbourhood associated with eating out behaviours and takeaway home delivery? A cross-sectional analysis of ProjectPLAN
Source: BMC Public Health. 2022 Jan 28;22:191. doi: 10.1186/s12889-022-12587-1 (PMC8796524; doi:10.1186/s12889-022-12587-1)

**Additional file 2** Participant flowchart for frequency outcomes.


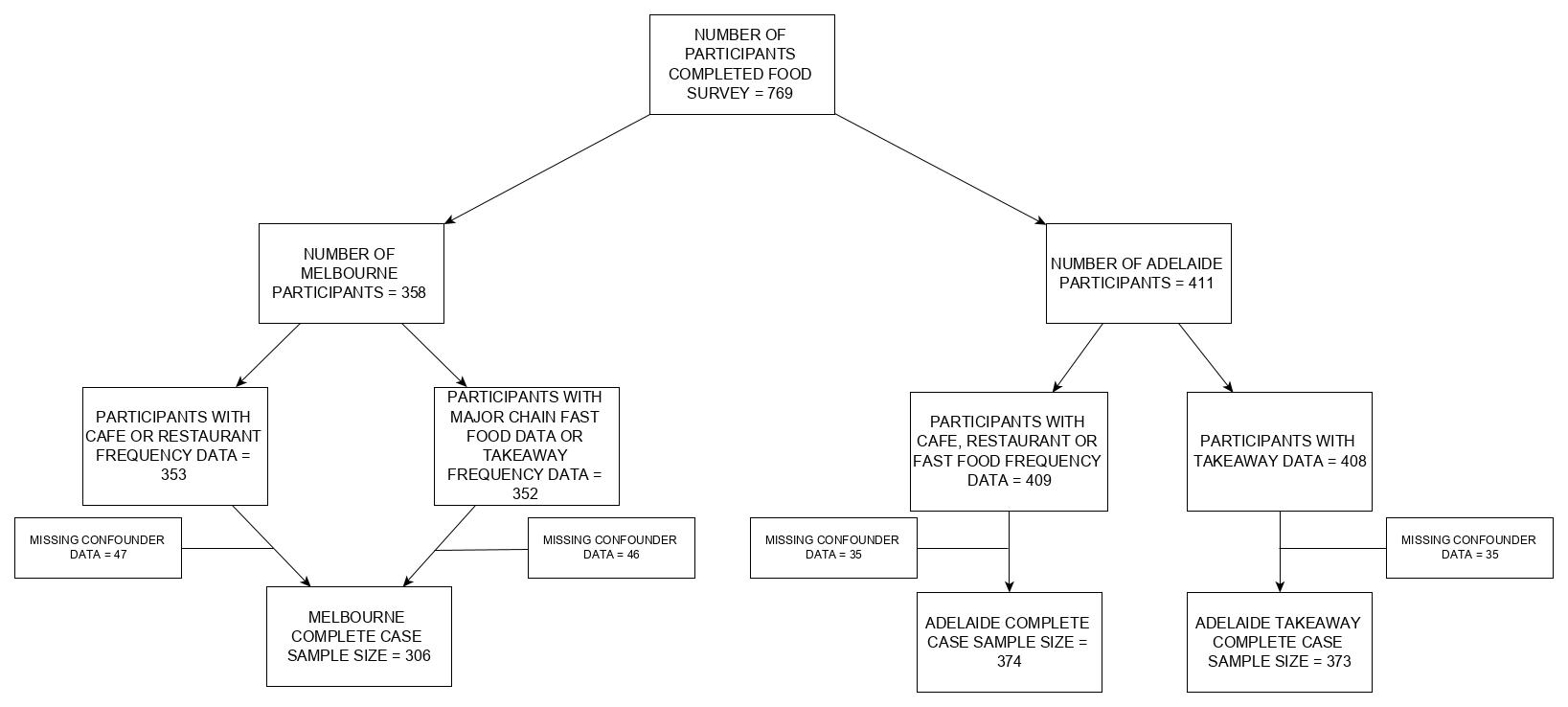

Supplement: Supplementary file 2 — Additional file 2. Participant flowchart for frequency outcomes. [file 12889_2022_12587_MOESM2_ESM.docx]

**Additional file 3.** Participant flowchart for distance outcomes.


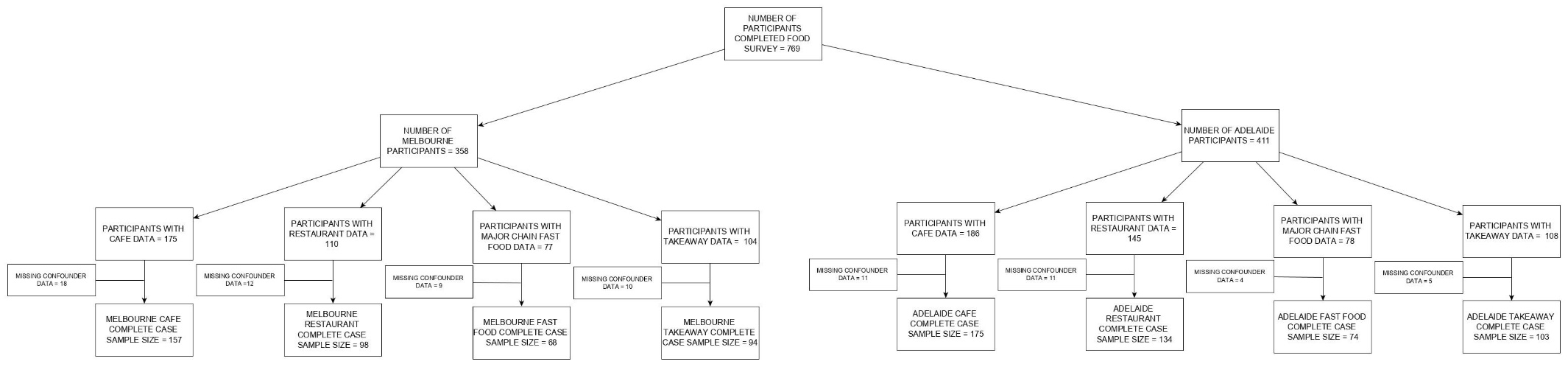

Supplement: Supplementary file 3 — Additional file 3. Participant flowchart for distance outcomes. [file 12889_2022_12587_MOESM3_ESM.docx]
