# Supplementary material for: Is having a 20-minute neighbourhood associated with eating out behaviours and takeaway home delivery? A cross-sectional analysis of ProjectPLAN
Source: BMC Public Health. 2022 Jan 28;22:191. doi: 10.1186/s12889-022-12587-1 (PMC8796524; doi:10.1186/s12889-022-12587-1)
Supplement: Supplementary file 4 — Additional file 4. Median and inter-quartile range of distances travelled to cafes, restaurants/bars/bistros, major chain fast food outlets and takeaway outlets by city and 20-min neighbourhood status*. [file 12889_2022_12587_MOESM4_ESM.docx]

**Additional file 4.** Median and inter-quartile range of distances travelled to cafes, restaurants/bars/bistros, major chain fast food outlets and takeaway outlets by city and 20-minute neighbourhood status*.

|  |  | **Melbourne** |  |  | **Adelaide** |  |
| --- | --- | --- | --- | --- | --- | --- |
|  | **Overall** | **20-minute neighbourhood** | **Non-20-minute neighbourhood** | **Overall** | **20-minute neighbourhood** | **Non-20-minute neighbourhood** |
| Distance to cafe visited most^1^ (km) | 3.6 (1.4, 7.7) | 1.8 (0.6, 6.3) | 4.7 (2.8, 8.3) | 3.3 (1.1, 8.7) | 1.4 (0.8, 4.6) | 6.3 (3.3, 17.1) |
| Distance to restaurant, bar or bistro visited most^2^ (km) | 3.8 (1.9, 7.4) | 2.2 (0.7, 5.2) | 5.0 (3.5, 11.1) | 3.6 (1.2, 9.0) | 1.8 (0.7, 4.6) | 6.7 (3.6, 14.5) |
| Distance to chain fast food outlet visited most^3^ (km) | 3.3 (1.9, 4.7) | 3.6 (1.5, 7.6) | 3.1 (2.0, 4.7) | 2.6 (1.5, 8.3) | 1.6 (1.2, 2.6) | 4.0 (2.7, 14.1) |
| Distance to takeaway outlet visited most^4^ (km) | 2.8 (1.5, 6.0) | 1.3 (0.5, 5.7) | 3.4 (2.2, 6.0) | 2.0 (1.2, 4.5) | 1.6 (0.9, 3.4) | 3.5 (1.9, 8.1) |

*Sample sizes differ for each outlet as not all participants regularly visit each of these outlets. ^1^Melbourne: N = 157; Adelaide: N = 175. ^2^Melbourne: N = 98; Adelaide: N = 134. ^3^Melbourne: N = 68; Adelaide: N = 74. ^4^Melbourne: N = 94; Adelaide: N = 103.
